# Boiling Technique-Based Food Processing Effects on the Bioactive and Antimicrobial Properties of Basil and Rosemary

**DOI:** 10.3390/molecules26237373

**Published:** 2021-12-04

**Authors:** Ahmad Mohammad Salamatullah, Khizar Hayat, Shaista Arzoo, Abdulhakeem Alzahrani, Mohammed Asif Ahmed, Hany M. Yehia, Tawfiq Alsulami, Nawal Al-Badr, Bandar Ali M Al-Zaied, Mohammed Musaad Althbiti

**Affiliations:** Department of Food Science & Nutrition, College of Food and Agricultural Sciences, King Saud University, Riyadh 11451, Saudi Arabia; asalamh@ksu.edu.sa (A.M.S.); sarzoo@ksu.edu.sa (S.A.); aabdulhakeem@ksu.edu.sa (A.A.); masifa@ksu.edu.sa (M.A.A.); hanyehia@ksu.edu.sa (H.M.Y.); talsulami@ksu.edu.sa (T.A.); nalbader@ksu.edu.sa (N.A.-B.); 437102343@student.ksu.edu.sa (B.A.M.A.-Z.); 437101927@student.ksu.edu.sa (M.M.A.)

**Keywords:** food processing, herbal tea, boiling, antioxidant activity, antimicrobial activity

## Abstract

Rosemary (*Rosmarinus officinalis*) and basil (*Ocimum sanctum* Linn) are mostly used as herbal teas, made by steeping whole or ground herbs in boiling water. Hence, it is important to know the effect of boiling time on the bioactivity of these herbs. The effect of different boiling times (5, 10, and 15 min) on the antioxidant and antimicrobial properties, and some selected phenolic compounds of these herbs was examined in this study. Experimental results revealed that basil displayed the highest total polyphenol content (TPC), total flavonoid content (TFC), and antioxidant activity when it was boiled for 5 min, and the lowest TPC was obtained when it was boiled for 15 min. On the other hand, rosemary had the highest TPC, TFC, and antioxidant potential after being boiled for 15 min, while it had the lowest after being boiled for 5 min. There was no growth inhibition of rosemary extracts against gram-negative bacteria, whereas higher growth inhibition was observed against gram-positive bacteria. The MIC and MBC of rosemary ethanolic extract against *Listeria monocytogenes* were 5 and 5 mg/mL and against *B. subtilis* were 10 and 10 mg/mL, respectively. While MIC and MBC of methanolic extract against *L. monocytogenes* were 5 and 5 mg/mL and against *Bacillus subtilis* were and 5 and 5 mg/mL, respectively. Salicylic acid was the most abundant (324.7 mg/100 g dry weight (dw)) phenolic compound in the rosemary sample boiled for 5 min, and acetyl salicylic acid was the most abundant (122.61 mg/10 g dw) phenolic compound in the basil sample boiled for 15 min.

## 1. Introduction

The reactive oxygen species (ROS) play a significant role in numerous cellular activities such as signaling transduction, gene transcription, and immune response [1]. ROS, either oxygen ions or oxygen-containing radicals are usually present at little concentrations [2]. The excess production of ROS either from the external sources or due to the endogenous metabolic processes in the human body causes oxidative damage to biomolecules resulting in several diseases such as neurodegenerative diseases, diabetes, cancers, chronic inflammatory diseases, and atherosclerosis [3,4,5]. The human body is equipped with antioxidants that counterbalance the harmful effects of oxidants as they are capable of scavenging the ROS and reducing the oxidation of cellular molecules [6].

Antioxidant-rich herbs serve as a great source of antioxidants in foods that strengthen the body’s ability to fight free radical damage and thus decreasing the risk of many diseases [7,8]. In addition to their content of antioxidants, herbs are well-known for their antimicrobial, antiseptic, diuretic, anti-inflammatory, analgesic, anthelmintic, and carminative properties [9,10]. Therefore, the lamiaceae family members including mint, thyme, basil, rosemary, sage, savory, and oregano are traditionally added to foods as flavors or used as medicines such as basil (*Ocimum sanctum* L.) and rosemary (*Rosmarinus officinalis* L.) [11].

Basil (*Ocimum sanctum* L.) is an aromatic herb and includes over 150 species, native to tropical areas of Africa, Asia, Central America, and South America [12]. It possesses stimulant and expectorant properties, anti-diabetic, anti-carcinogenic, anti-inflammatory, and antimicrobial characteristics. The properties of basil make it traditionally used to manage the multiple medical conditions such as chest complications, cough, bronchitis, stress, anxiety, gastritis, dysentery, skin diseases, asthma, diarrhea, fever, arthritis, and eye diseases [13,14,15,16,17,18,19,20,21]. Basil also guards against the lethal effects of industrial chemicals and many pharmaceutical drugs [22]. Basil herbs are rich source of vitamin A, C, and minerals including calcium, iron, and zinc [23]. The leaf volatile oil contains eugenol, eugenol, ursolic acid, carvacrol, methyl carvicol, linalool, and sitosterol [19,24]. The presence of these components depends on the basil species, weather, location and growing conditions, and growth period/level during harvest [25,26,27,28]. Similarly, rosemary (*Rosmarinus officinalis* L.) is also one of the most popular perennial culinary herbs of the lamiaceae family. This plant comes from the Mediterranean region and is cultivated all over the world. In folk medicine, rosemary is used to control numerous diseases such as headache, stomachache, dysmenorrhea, epilepsy, depression, nervous agitation, rheumatic pain, fatigue, spasms, and improvement of memory. In addition, it possesses antioxidant, antimicrobial, anti-inflammatory, anti-apoptotic, anti-tumorigenic, antinociceptive, and neuroprotective properties [29,30]. Rosemary provides protein, vitamins, minerals, and fiber which are known to have disease preventing properties [31]. It has many phytochemicals which constitute natural compounds as phenolic diterpenes, flavonoids, and phenolic acids. The main constituents of the rosemary essential oil are 1,8-cineole, camphor, α-pinene, camphene, borneol, β-pinene, and limonene [32,33,34]. Tawfeeq et al., [35] and Jiang et al., [36] stated 1,8-cineole to be the main component of rosemary essential oil while Bendeddouche et al., [37] reported camphor followed by 1,8-cineole to be the main components of rosemary essential oil. This difference may be due to the vegetative stage and bioclimatic conditions [33]. The most common polyphenols, the secondary metabolites of rosemary are apigenin, homoplantaginin, diosmin, gallocatechin, luteolin, and genkwanin. Apart from this, it also contains various phenolic acids such as chlorogenic, rosmarinic, and caffeic acid [38,39,40]. In a study on antioxidant and antibacterial properties of some fresh and dried Labiatae herbs, the fresh and commercial rosemary had the highest antioxidant activity and phenolic content, but oven-dried rosemary ranked third [41]. The reduction in antioxidant values following thermal treatments has been credited to the enzymatic degradation of phenolic compounds, thermal degradation of phytochemicals, and loss of antioxidant enzyme activities [42]. The strong antioxidant properties of the commercial brand of rosemary may be due to freeze-drying in which heat is not involved [41]. In contrast to this, other studies have observed an increase in antioxidant activity and phenolic content following the thermal treatment and suggested that phenolic compounds may be released through the breakdown of cellular constituents and formed new compounds with increased antioxidant capacity [43,44].

In several Asian nations, basil and rosemary as therapeutic herbs are prepared in the traditional form of herbal teas by extricating whole or ground herbs in bubbling water. Despite the availability of numerous studies examining the antioxidant properties of basil and rosemary, hardly any research has been published evaluating the effect of boiling conditions on their antioxidant contents. Consequently, the purpose of this study was to determine the possible effects of boiling on the total polyphenol and flavonoid contents, antioxidant activity, and antimicrobial proprieties of basil and rosemary leaves at various times.

## 2. Results and Discussion

### 2.1. Effect of Boiling on Total Polyphenol Content of Basil and Rosemary

The effect of boiling time on the total polyphenol content (TPC) of basil and rosemary is provided in Table 1. It clearly shows that the boiling time significantly affected the TPC of both samples. The highest TPC of basil was achieved by boiling it for 5 min while the lowest TPC was obtained when the boiling time was 15 min. It also showed that the boiling time had an adverse effect on the phenolic compounds of basil. In a recent study, it was noticed that the boiling process caused a reduction in the total phenol content and the antioxidant activity of the celery roots [45]. However, the TPC content of the rosemary showed an increasing trend with the increase in the boiling time. For example, the TPC of the rosemary sample boiled for 5 and 15 min was 122.84 and 140.43 mg, respectively, gallic acid equivalent (GAE) per gram dry weight (dw). This may be due to the heat during boiling, which may rupture the cell wall of the material causing the release of phenolic compounds in the solvent [46,47]. Our results are in line with a recent study where the boiling process of green and red rooibos (Aspalathus linearis) herbal tea delivered a higher TPC and antioxidant activity [48]. In brief, rosemary exhibited a significantly (*p* < 0.05) higher TPC than the basil in all analyzed samples, which can be attributed to the phenolic compounds content and antioxidant potential of the plants itself [49,50].

### 2.2. Effect of Boiling on Total Flavonoid Content of Basil and Rosemary

As shown in Table 1, the total flavonoid content (TFC) of basil was decreased by increasing the boiling time. A total flavonoid content of 39.66 and 36.79 mg catechin equivalent (CE) per gram dry weight was obtained for the basil samples boiled for 5 and 15 min, respectively. An earlier study reported a loss of phenolic compounds and the antioxidant activity on boiling for 10 min of various green vegetables [51]. In contrary to the basil, the total flavonoid content of rosemary was increased significantly by increasing the boiling time. The highest flavonoid content (109.73 mg CE/g dw) was noted for the rosemary sample boiled for 15 min, while 5 min of boiling provided a flavonoid content of 122.84 mg CE/g dw. In a recent study, it was reported that the boiled edible leaves of *Sesbania grandiflora*, *Cassia auriculata*, *Centella asiatica*, and *Gymnema lactiferum* showed an increase in the total content of phenols and flavonoids compared with the fresh ones [49]. Similar to the TPC, the TFC of all the rosemary samples was significantly higher than those of the basil samples.

### 2.3. Effect of Boiling on DPPH Scavenging of Basil and Rosemary

The DPPH scavenging of basil and rosemary in terms of their 50% inhibitory concentration (IC_50_) is provided in Table 1. The higher the IC_50_ value, the lower is the antioxidant potential of the sample. For basil samples, 5 min of boiling significantly resulted in the lowest IC_50_ value (6.08 mg) compared with the samples boiled for 10 min (6.39 mg) and 15 min (6.62 mg), respectively. For the rosemary sample, the lowest IC_50_ value, the highest DPPH scavenging potential, was achieved at 15 min of boiling. A study conducted by Gunathilake et al., [49] showed that the boiled edible leaves of C. auriculata, Passiflora edulis, and C. asiatica resulted in a higher DPPH scavenging compared with their fresh counterparts. However, Arias-Rico et al., [52] revealed that the DPPH scavenging and polyphenols of the leaves and stems of *Chenopodium nuttalliae* Safford, *Suaeda torreyana* S. Watson, *Portulaca oleracea* L., *Chenopodium album* L., and *Porophyllum ruderale* (Jacq.) were decreased by boiling either for 3 or 5 min. The rosemary showed significantly (*p* < 0.05) higher DPPH scavenging compared with the basil. These results echoed the TPC and TFC results showing that the DPPH potential of the samples was due to at least a part of their TPC and TFC contents.

### 2.4. Effect of Boiling on Ferric Reducing Power of Basil and Rosemary

The effect of boiling on the ferric reducing power of basil and rosemary is provided in Table 1. The reducing power of basil boiled for 5 min was significantly (*p* < 0.05) higher (0.815) compared with the samples boiled for 10 min (0.789) and 15 min (0.712). For the rosemary sample, 15 min of boiling provided the highest reducing power while 5 min of boiling exhibited the lowest reducing power. Nie et al. [53] assessed the effect of boiling time (10–120 min) on the nutritional value and the antioxidant capacity of a mushroom Lentinus edodes. They found that the antioxidant capacity was increased during the first 30 min of boiling and then kept stable or declined on prolongation of the boiling time. Overall, the rosemary showed higher reducing power than that of the basil extracts, which are in an agreement with the results of TPC and TFC.

### 2.5. Antimicrobial Activity

**Figure 1:**
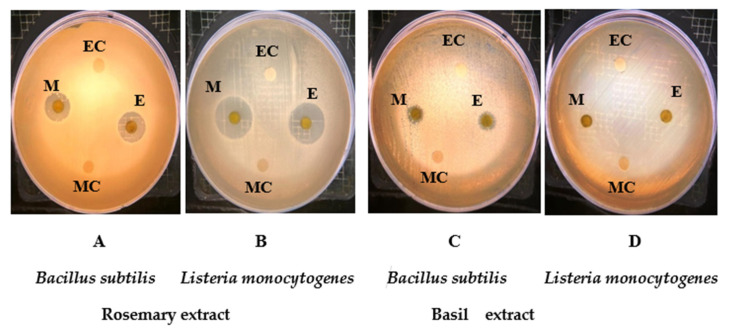
Zone of inhibition for both ethanolic and methanolic extracts of rosemary and basil against *B. subtilis* (**A**,**C**) and *L. monocytogenes* (**B**,**D**). E: Ethanolic extract of rosemary; M: Methanolic extract of rosemary; EC: Ethanol control; MC: Methanol control.

Both ethanolic and methanolic rosemary extracts had no effect on the gram-negative bacteria (*Escherichia coli* and *Salmonella typhimurium*), but they affected the gram-positive bacteria (*L. monocytogenes* and *B. subtilis*) (Figure 1A,B). Both extracts inhibited the growth of *L. monocytogenes* and provided a zone of inhibition ranging from 15 to 20 mm in diameter (Table 2). Similar zones of inhibition were observed with *B. subtilis* using ethanol and methanol extracts. The zones of inhibition exhibited a diameter of 14 and 15 mm with ethanol and methanol, respectively (Table 2). In comparison with rosemary, basil extracts in ethanol or methanol showed no effect on the tested pathogens except for *B. subtilis*, which was partially inhibited (with the zone of inhibition being less than 8 mm in diameter) (Figure 1C). This step was carried out using 50 μL of rosemary extract (100 mg/mL) to determine if there was an effect against the tested gram-positive bacteria. The effect of cold and hot aqueous extract of rosemary has shown a high inhibition rate against *Proteus* sp., *Klebsella* sp., *E. coli*, and *Pseudomonas* sp., because it contains a number of hydroxyl groups that act as a hydrogen donor making it very important and powerful [54].

The MIC of the ethanolic and methanolic rosemary extracts against *L. monocytogenes* was 5 and 5 mg/mL, respectively, and against *B. subtilis* were 10 and 5 mg/mL, respectively. Similarly, the MBC of the ethanolic and methanolic rosemary extracts against *L. monocytogenes* were 5 and 5 mg/mL respectively, and against *B. subtilis* were 10 and 5 mg/mL respectively. It was revealed that when methanol was used as a solvent to extract rosemary, there was no growth of both *L. monocytogenes* and *B. subtilis* on Mueller–Hinton agar, indicating that rosemary extraction in methanol has a bactericidal effect against both pathogenic bacteria [55]. On the other hand, the ethanolic extract of rosemary had bactericidal effect only against *L. monocytogenes*. In a study conducted by Gonelimali et al. [56], it was revealed that the ethanolic and water extract of selected plants (roselle, rosemary, clove, and thyme) efficiently suppressed the growth of food pathogens and spoilage microorganisms with variable potency. The ethanolic extract of rosemary exhibited an inhibitory effect against four of the pathogenic strains (*E. coli*, *Salmonella enteritidis*, *Bacillus cereus*, and *Salmonella aureus*), while aqueous extract of rosemary was effective against all strains except *S. enteritidis*.

The antimicrobial activity of rosemary depends mainly on its phenolic and flavonoid content [57]. It cannot be dissolved in water, but in drought conditions it can reduce its resistance [58]. Leaves of rosemary, either fresh or dried, are aromatic and usually added in small quantities for cooking to improve taste. Due to its antioxidant content, rosemary extract is also used as a natural preservative to extend the shelf life of perishable foods according to the UK Food Standards Agency [59]. Thus, the evidence indicates that the antioxidant content of rosemary may help in fighting the bacterial infections and serious diseases such as cancer. The immune-boosting properties of rosemary have also been shown to help in defense against Helicobacter pylori, which causes gastric ulcers, and Staphylococcus infections, which causes a wide variety of clinical diseases [60]. A variety of factors can affect the antioxidant content of rosemary, such as the plant quality, the geographical origin, the date of harvest, the method of extraction, and even the climatic conditions where the plant was grown [61].

### 2.6. HPLC Analysis of Phenolic Compounds

Results of phenolic compounds analyzed by HPLC are reported in Table 3. Chlorogenic acid was higher in the rosemary sample boiled for 15 min (70.61 mg/100 g dw) compared with those boiled for 5 and 10 min. The rosemary sample boiled for 5 min, however, contained the highest concentration of salicylic acid (324.7 mg/100 g dw). The quercetin was only detected in the rosemary sample and its highest amount (243.64 mg/100 g dw) was found in the sample boiled for 15 min. The phenolic compounds content in the rosemary sample boiled for 10 min ranged from 3.78 to 137.10 mg/100 g dw. Moreover, vanillin (2.29 mg/100 g dw) was found in the lowest amount in the rosemary sample boiled for 5 min. The total phenolic compounds were higher (808.38 mg/100 g dw) in the rosemary sample boiled for 15 min, followed by the 5 min (746.12 mg/100 g dw) and 10 min boiled sample (529.03 mg/100 g dw).

In basil, the highest phenolic compound was acetyl salicylic acid (122.61 mg/100 g dw) then chlorogenic acid (20.67 mg/100 g dw) in the 15 min boiled sample. While, with respect to other compounds, 3,5 dinitro salicylic acid was the lowest (0.42 mg/100 g dw) phenolic compound in the basil sample. Total phenolic compounds were higher (163.06 mg/100 g dw) in the 15 min boiled basil sample, compared with the 5 min and 10 min boiled samples which contained 117.99 mg/100 g dw and 111.14 mg/100 g dw, respectively.

The HPLC results of rosemary were in accordance with the results provided in Table 1. Total polyphenol content and reducing power (Table 1) were increased at 15 min of boiling for the rosemary sample, similarly the phenolic compounds were high at 15 min of boiling (808.37 mg/100 g dw) as quantified by HPLC. While, for the basil sample, the total polyphenol content and reducing power decreased with the increase in the boiling time (Table 1) in contrast to the HPLC results where the phenolic compounds were increased at 15 min of boiling compared with 5 min of boiling of the basil sample. It showed that there might be some other compounds (except detected by HPLC in this study) contributing to the antioxidant activity of the basil sample. Elansary et al. [62] revealed that the caffeic acid was 27.6 mg/100 g dw in methanolic rosemary extract. Furthermore, Flanigan et al. [63] reported that caffeic acid was 19.9 mg/100 g dw in basil. Begum et al. [33] reported the phenolic acids (rosmarinic, chlorogenic and caffeic acid) were >3% in rosemary samples.

## 3. Materials and Methods

### 3.1. Raw Materials

The raw material basil (*Ocimum sanctum* L.) was collected from a vegetable garden in King Saud University, Saudi Arabia, while the rosemary (*Rosmarinus officinalis*) was acquired from the local market in Riyadh, Saudi Arabia. The samples were cleaned, washed with water, cut into small pieces, and dried overnight in an oven dryer at 40 °C. The samples were ground using a coffee grinder and sieved with stainless steel wire mesh (25 mm). Powdered samples were stored at −20 °C in an airtight container until used.

### 3.2. Preparation of Phenolic Extracts

Two grams (2.0 ± 0.05 g) of powdered samples were extracted with 20 mL of nano-pure water. The suspension was heated to the boiling point, and results were observed at 5, 10, and 15 min. The boiling time was selected based on the results of pre-trials. The pre-trials were conducted for different boiling times and data were obtained for TPC and the reducing power. The boiling times which were suitable for both the basil and the rosemary were selected for this study. Once the boiling was finished for their respective time, the mixture was cooled at room temperature. The obtained mixture was then centrifuged at room temperature for 10 min at 3000× *g* (HERMLE Labortechnik GmbH. Siemensstr. 25 D-78564 Wehingen, Germany) and filtered using Whatman filter paper number 2. The obtained extracts were stored at 4 °C and used for analyses.

### 3.3. Total Polyphenol Content (TPC)

The Folin–Ciocalteu (FC) method was followed to detect TPC [64]. Initially, 125 µL of undiluted FC reagent was added to 25 µL of extract. Subsequently, the mixture was shaken for 1 min at room temperature after the addition of 1.5 mL of nano pure water. After 1 min of shaking, 375 µL of 20% sodium carbonate and 475 µL of water were added to the mixture and the final volume was made to 2500 µL. TPC detection was achieved spectroscopically at 760 nm (Jasco, V-630 spectrophotometer, Easton, MD, USA) after 30 min of incubation at room temperature. The TPC was expressed as the gallic acid equivalent per gram dry weight of the sample (mg GAE/g dw).

### 3.4. Total Flavonoid Content (TFC)

In the current study, the TFC was measured according to the procedure suggested by Hayat [64]. A total of 1 mL of water was added to 250 µL of extract. Then, 75 µL of each 5% (*w*/*v*) sodium nitrite and 10% (*w*/*v*) aluminum chloride were added. The mixture was allowed to stand at room temperature for 5 min. After that, 0.5 mL of sodium hydroxide (1 M) and 0.6 mL of water were added to the mixture and then vortexed. Blank samples were prepared following the same steps but without extract. Total flavonoid detection was achieved spectroscopically at 510 nm (Jasco, V-630 spectrophotometer, USA). TFC was expressed as the mg catechin equivalent per gram dry weight of the sample (mg CE/g dw).

### 3.5. DPPH Scavenging

The free radical scavenging capacity of the basil and rosemary extract was analyzed using DPPH according to the method suggested by Gülçin et al. [65] with slight modifications. Firstly, 130 µL aliquot of extract was mixed with 0.1 mM DPPH and then incubated in the dark for 30 min. The absorbance was measured at 510 nm (Jasco, V-630 spectrophotometer, USA). Control samples were prepared following the same steps, however, ethanol, as a blank, was added to the control samples instead of extract. The scavenging percentage was calculated as Equation (1):

DPPH scavenging % = Acontrol − Asample/Acontrol × 100(1)

### 3.6. Reducing Power

The ferric reducing power of the sample was analyzed according to the steps used by Hayat et al., [66]. Initially, potassium ferricyanide (1.25 mL) was mixed with the extract (0.5 mL). Then, 1.25 mL of sodium phosphate buffer (0.2 M, pH 6.6) was added to the mixture and incubated for 20 min at 50 °C. Subsequently, 1.25 mL of trichloroacetic acid was added and then centrifuged at 3000× *g* for 10 min at room temperature. Lastly, 1.25 mL of water and 0.25 mL of ferric chloride were added to 1.25 mL of aliquot from the supernatant. Blank samples were prepared without extract and the absorbance was recorded at 700 nm (Jasco, V-630 spectrophotometer, USA).

### 3.7. Bacterial Strains

The standard reference strains of American Type Culture Collection (ATCC): *Listeria monocytogenes* ATCC 19114 (Microbiologics Inc., St. Cloud, MN, USA), *Bacillus subtilis* ATCC 6633 (Microbiologics Inc., St. Cloud, MN, USA), *Escherichia coli* ATCC 10798 (Microbiologics Inc., St. Cloud, MN, USA), and *Salmonella typhimurium* ATCC 14028 (Microbiologics Inc., St. Cloud, MN, USA), were used in this study.

### 3.8. Antibiotic Proprieties of Basil and Rosemary

An agar diffusion assay [67] was performed in order to assess the antimicrobial efficiency of the basil and rosemary extracts against the foodborne pathogens listed previously. Active bacterial strains were inoculated in Mueller–Hinton broth (Oxoid, CM0405) at a concentration of 10^6^ CFU/mL and incubated overnight. From this broth, 100 µL was spread-plated onto Mueller–Hinton agar (Oxoid CM 0337). The basil and rosemary extracts were dissolved separately in 96% ethanol and methanol to provide a final concentration of 100 mg/mL and all samples were stored at 4 °C until further analysis. Three sterilized 6 mm Whatman filter paper disks were saturated as follows: the first disc was saturated with 50 µL of methanolic basil extract, while the second disc was saturated with 100 µL of the same substance. For a control, the disc was saturated with only methanol. The same procedure was followed with the methanolic rosemary extract, as well as the ethanolic extracts of both herbs (using ethanol on the control disc instead). The inhibition zone (mm) was determined for each tested pathogen with each extract after incubation at the suitable temperature.

### 3.9. Determination of Minimum Inhibitory and Minimum Bactericidal Concentrations

Each colony of the four tested bacteria were inoculated into Mueller–Hinton broth (Oxoid, CM0405) and incubated at 37 °C for 24 h. The bacterial culture was adjusted to ~0.5 optical density at 650 nm using a spectrophotometer (GloMax^®^ Multi), corresponding to a concentration of 1 × 10^8^ CFU/mL (0.5 McFarland standard). One hundred microliters of ethanolic and methanolic extracts of basil were separately dispersed into two a 96-well flat-bottom plate (Costar^®^) and mixed well with 100 µL of bacterial inoculum. The optical density was measured after a 24 h incubation period [68,69].

A microdilution assay was performed to assess the MIC of rosemary and basil extracts according to the procedure suggested by Andrew [69]. First, 100 μL of Mueller–Hinton broth (Oxoid, CM0405) was distributed onto the microtiter plate. Then, 100 μL of the test extracts (basil and rosemary at a concentration of 100 mg/mL) were dispensed into to the first well of the microtiter plate and serially diluted. A 100 μL aliquot of the bacterial inoculum (~5 × 10^5^ CFU/mL) was mixed well with each extract dilution. The plates were incubated at 37 °C for 24 h with shaking before determining the optical density of the bacterial growth. The MIC was recorded as the lowest concentration of the test agent that resulted in bacterial growth inhibition [69].

The MBC of the tested substances was derived from the positive density recorded from the MIC results. Therefore, 10 μL was pipetted and sub-cultured onto the Mueller–Hinton agar and incubated for 24 h at 37 °C [70]. The MBC was determined to be the concentration, at which no visible growth of bacteria appeared on the cultured plate, i.e., the lowest concentration of substance required to achieve the bactericidal killing [68].

### 3.10. HPLC Analysis of Phenolic Compounds

Phenolic compounds (chlorogenic acid, acetyl acetic acid, tannic acid, quercetin, resorcinol, caffeic acid, 1,2-dihydroxybenzene, vanillin, 3,5-dinitrosalicylic acid (3,5-DNSA), and salicylic acid) in basil and rosemary samples were analyzed with HPLC according to the method of He et al. [71] with some modification. The Shimadzu (prominence) HPLC system (Kyoto, Japan) equipped with binary pump LC-20 AB, UV detector SPD-10 A was used in this work. Phenolic compounds were separated on the column Zorbax SB-C_18_ (250 × 4.6 mm, 5µm) with mobile phase, (A) 1% acetic acid and (B) 100% methanol, and detected at wavelength 280 nm. The binary gradient program for the mobile phase with 1 mL/min flow rate was 0–10 min for 15–30% (B); 10–20 min for 30–40% (B); 20–30 min for 40–50% (B); 30–41 min for 50–60% (B), and 41–45 min for 15% (B). Phenolic compounds in basil and rosemary samples were compared with the retention time of standards. All samples were analyzed in duplicate and the arithmetical mean ± standard error was reported.

### 3.11. Statistical Analysis

The statistical analysis was performed using SAS (v9.2, 2000–2008; SAS Institute Inc., Cary, NC, USA) for data analysis. All the tests were performed in triplicates and the results were presented as mean ± standard deviation (SD). A one-way analysis of variance (ANOVA, *p* ≤ 0.05) was used to analyze the differences among the treatment groups and a post-hoc analysis using Duncan’s multiple range tests was performed if any significant differences were found.

## 4. Conclusions

The duration of boiling time has a significant influence on the medicinal properties of traditional herbs. Boiling for 5 min showed the higher TPC, TFC, and antioxidant activity of the basil samples, while 15 min of boiling exhibited better results for rosemary samples. Overall, the rosemary extracts were effectively better than the basil extracts with respect to TPC, TFC, and antioxidant capacity. The MIC and MIB of rosemary ethanolic extract against *L. monocytogenes* were 5 and 5 mg/mL, respectively, and against *B. subtilis* were 10 and 10 mg/mL, respectively. Similar zones of inhibition were noted against *B. subtilis* and were 14 and 15 mm in diameter using ethanolic and methanolic extracts, respectively. Basil extracts in either ethanol or methanol had no effect on the same tested microorganisms in comparison with rosemary except against *B. subtilis*. While the MIC and MBC of methanolic extract for *L. monocytogenes* and *B. subtilis*, were 5 mg/mL, respectively. According to the HPLC results, among the tested compounds, acetyl salicylic acid was obtained in the highest phenolic compound in the basil sample boiled for 15 min, while, salicylic acid was the highest phenolic compound in the rosemary sample boiled for 5 min. The results of this study might be helpful to reap the maximum health benefits of the teas produced from these herbs. In addition, as the response surface methodology (RSM) is one of the most commonly used experimental designs for the optimization of process conditions, so based on this study, the RSM approach in the near future is also suggested to determine the optimum conditions for the extraction process.

## Figures and Tables

**Table 1:** Effect of boiling on the bioactive properties of basil and rosemary.

Sample	Process Time	Total Polyphenol Content (mg GAE/g dw)	Total Flavonoid Content (mg CE/g dw)	DPPH (IC_50_ mg/g)	Ferric Reducing Power (Absorbance 760 nm)
Basil	5 min	69.24 ± 1.03 ^c^	39.66 ± 0.08 ^d^	6.08 ± 0.15 ^b^	0.815 ± 0.012 ^c^
	10 min	66.22 ± 3.89 ^c^	39.00 ± 0.63 ^d,e^	6.39 ± 0.15 ^a^	0.789 ± 0.102 ^c,d^
	15 min	54.64 ± 6.13 ^d^	36.79 ± 0.26 ^e^	6.62 ± 0.27 ^a^	0.712 ± 0.009 ^d^
Rosemary	5 min	122.84 ± 5.79 ^b^	78.36 ± 1.55 ^c^	0.79 ± 0.01 ^c^	1.426 ± 0.013 ^b^
	10 min	119.24 ± 2.47 ^b^	86.85 ± 2.80 ^b^	0.82 ± 0.03 ^c^	1.503 ± 0.040 ^a,b^
	15 min	140.43 ± 4.44 ^a^	109.73 ± 0.33 ^a^	0.66 ± 0.01 ^d^	1.526 ± 0.037 ^a^

The values for each assay are expressed as mean ± standard deviation of three replicates. Different superscript letters in the same column represent the significant differences in data (*p* < 0.05).

**Table 2:** Zone of inhibition (mm), MIC and MBC of the of ethanolic and methanolic extracts of rosemary against *L. monocytogenes* and *B. subtilis*.

	Plant Extract	Rosemary Extract							
Microorganisms		Ethanolic Extract Rosemary				Methanolic Extract Rosemary			
		Zone of Inhibition (mm)	MIC (mg/mL)	MBC (mg/mL)	Growth on MH Agar	Zone of Inhibition (mm)	MIC (mg/mL)	MBC (mg/mL)	Growth on MH Agar
*L. monocytogenes* ATCC 19114		20	5	5	NG/Bactericide	15	5	5	NG/Bactericide
*B. subtilis* ATCC 6633		14	10	10	+++/Bacteriostatic	15	5	5	NG/Bactericide
*S. typhimurium* ATCC 14028		-	-	-	-	-	-	-	-
*E. coli* ATCC 10798		-	-	-	-	-	-	-	-

NG: no growth; -: no effect., +++: good growth; ATCC: American Type Culture Collection

**Table 3:** Effect of boiling on phenolic compounds of rosemary and basil by HPLC (mg/100 g) dry weight (dw).

Sample	Process Time	Resorcinol	Chlorogenic Acid	Caffeic Acid	Vanillin	Acetyl Salicylic Acid	3,5-DNSA	Salicylic Acid	Quercetin	Total
Basil	5 min	ND	20.38 ± 0.15	2.03 ± 0.23	1.64 ± 0.04	63.58 ± 0.83	24.87 ± 1.48	5.49 ± 0.09	ND	117.99 ± 2.82
	10 min	ND	19.23 ± 0.56	4.36 ± 0.67	16.52 ± 0.23	48.18 ± 0.67	19.32 ± 0.67	3.53 ± 0.68	ND	111.14 ± 2.81
	15 min	ND	20.67 ± 0.69	2.72 ± 0.51	15.20 ± 0.35	122.61 ± 0.58	0.42 ± 0.35	1.44 ± 0.92	ND	163.06 ± 3.40
Rosemary	5 min	4.84 ± 0.26	25.56 ± 2.07	14.41 ± 1.38	2.28 ± 0.66	165.35 ± 1.21	42.99 ± 1.80	324.70 ± 2.50	165.99 ± 2.15	746.12 ± 12.03
	10 min	5.77 ± 0.28	5.76 ± 0.05	51.50 ± 1.32	3.78 ± 1.41	135.79 ± 3.62	87.17 ± 1.17	102.16 ± 1.11	137.10 ± 1.40	529.03 ± 10.36
	15 min	5.28 ± 0.08	70.61 ± 0.37	14.63 ± 0.44	2.35 ± 0.37	161.81 ± 1.17	143.15 ± 0.78	166.91 ± 3.68	243.64 ± 0.61	808.38 ± 7.50

ND: Not detected.

## Data Availability

The data presented in this study are available on request from the corresponding author.

## Sample Availability

Samples of the compounds are not available from the authors.
